# Proteoglycans as Therapeutic Targets in Brain Cancer

**DOI:** 10.3389/fonc.2020.01358

**Published:** 2020-08-05

**Authors:** Zoya Yan, Shanzhi Wang

**Affiliations:** ^1^Horace Greeley High School, Chappaqua, NY, United States; ^2^Chemistry Department, University of Arkansas at Little Rock, Little Rock, AR, United States

**Keywords:** proteoglycan, glycosaminoglycan, glioblastoma, brain cancer, biomarker, inflammation

## Abstract

Proteoglycans (PGs) are heavily glycosylated diverse proteins consisting of a “core protein” covalently attached to glycosaminoglycans (GAGs) and present on the cell surface, extracellular matrix, and intracellular milieu. Extracellular proteoglycans play crucial roles in facilitating cell signaling and migration, interacting with growth factor receptors, intracellular enzymes, extracellular ligands, and matrix components, as well as structural proteins and promoting significant tumor-microenvironment interactions in cancerous settings. As a result of their highly regulated expression patterns, recent research has focused on the role of proteoglycans in the development of nervous tissue, such as their effect on neurite outgrowth, participation in the development of precursor cell types, and regulation of cell behaviors. The present review summarizes current progress for the studies of proteoglycan function in brain cancer and explains recent research involving brain glycoproteins as modulators of migration, cell adhesion, glial tumor invasion, and neurite outgrowth. Furthermore, we highlight the correlations between specific proteoglycan alterations and the suggested cancer-associated proteoglycans as novel biomarkers for therapeutic targets.

## Introduction of Brain Cancer

Brain tumors are abnormal cell growths in the brain, though only malignant tumors are cancerous. There are two types of brain tumors: the primary brain tumor, which originates from and resides within the brain, and the secondary (metastatic) brain tumor, which originates from cancer outside of the central nervous system (CNS) then spreads into the brain. While primary tumors are the more frequent solid tumors in children, metastatic tumors are more commonly diagnosed in adult patients (1).

In recent decades, the worldwide brain tumor incidence rate has increased across all ages. The standardization of age in varying countries is between 0.01–12.7 in males and 0.01–10.7 in females per 100,000 people, with the highest incidence in northern Europe and the lowest in Africa (2). According to the Central Brain Tumor Registry of the United States (CBTRUS), the incidence rate of CNS tumors in the United States (23.03 per 100,000 cases for a total count of 392,982) incident tumors, of which 121,277 cases are malignant and 271,705 non-malignant) is lower in males (20.59 per 100,000 for 165,148 total cases) than in females (25.31 per 100,000 for 227,834 total cases) (3). In addition, in the United States, the 5-years survival rate following the diagnosis of a primary malignant CNS tumor is about 35% (2008–2014 data)^1^. The mortality rate of CNS cancers is estimated at about 3.4 per 100,000 in the world (2).

Across all age groups, the most common brain tumors develop from glial cells, which are gliomas that encompass a large scope of tumors and can be classified into four grades as follows: grade I (pilocytic astrocytoma), grade II (diffuse astrocytoma), grade III (anaplastic astrocytoma), and grade IV (glioblastoma multiforme) (4). Grade III and IV are categorized as high-grade or malignant gliomas with extremely poor prognosis, with grade IV diagnoses (which account for half of primary brain tumors) seeing a 5-years survival rate of <10% (5). The most common intracranial tumors in adults are brain metastases, with over 150,000 cases in the United States alone. Despite the considerable effect of varying primary tumor types on the incidence of metastases, 8–10% of adults diagnosed with cancer will develop brain metastases (6).

Developing treatment for CNS cancer is one of the most exigent branches of study in oncology. Although therapeutic approaches that exploit the immune system are a promising alternative strategy to surgery, radiotherapy, and anticancer drug therapy, multidrug resistance is a substantial obstacle restricting the success of conventional chemotherapy (7). The innate chemoresistance of many primary brain tumors and insufficient penetration of cytotoxic drugs across the blood-brain barrier (BBB) are also both responsible for the unsuccessful response of brain tumors to chemotherapy (8). Due to the urgency for novel therapies that combat these occurrences, researchers have emphasized and prioritized the development of anticancer drugs.

Using *in vitro* methods, Kwon et al. directly tested the effects of sialic acid glycan and glycosylation on BBB influx and efflux of IgG, specifically the influx and efflux processes for BBB endothelial cells, and facilitated “direct measurement of the Permeability Coefficient in each direction” (9, 10). In the study, BBB pharmacokinetics were found to be considerably affected by modest changes of IgG glycan profiles with sialylated glycans, suggesting that modifying IgG glycan could become an effective technique in increasing the concentration of the brain's therapeutic antibodies. Because immune pathways induced by sialylated IgG cause little inflammation, sialylation may therefore suggest beneficial clinical possibilities and further implications for patients with other CNS diseases such as Alzheimer's disease (11–13).

## Roles of Proteoglycans in Brain Cancer

**Figure 1:**
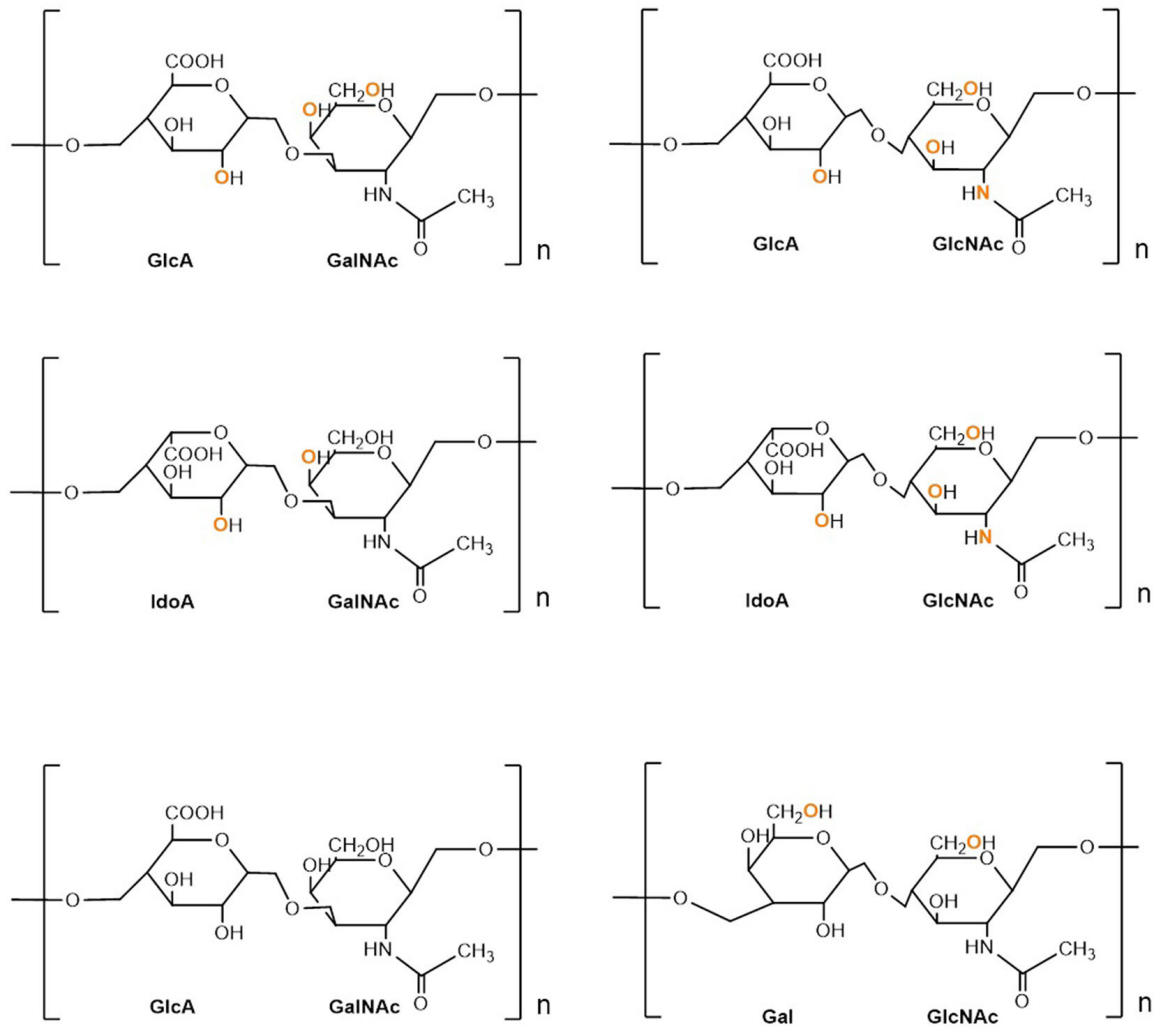
Structure and composition of GAGs. Sulphation sites are shown in orange.

Proteoglycans (PGs) are heavily glycosylated proteins and present on the cell surface, extracellular matrix (ECM), and intracellular milieu (14). The basic PG unit consists of a “core protein” covalently attached to glycosaminoglycans (GAGs), which are long chains consisting of linear or branched carbohydrate polymers that are negatively charged under certain physiological conditions, expressed on most mammalian cells. The six known GAGs are heparin (HP), hyaluronic acid (HA), heparan sulfate (HS), chondroitin sulfate (CS), keratan sulfate (KS), and dermatan sulfate (DS). These GAGs' respective disaccharide units contain different uronic and amino sugars (15). Their structure is demonstrated in Figure 1. GAGs are considerably linked to several diseases, including cancer, inflammation, bacterial infections, multiple sclerosis, viral infections, and Alzheimer's disease, as they interact with ligands to modulate physiological and pathological processes. Given such active involvement, GAG-based drugs are of considerable interest to researchers and have yielded promising outcomes in both animal and clinical trials, suggesting prospective development in therapeutics (16).

**Figure 2:**
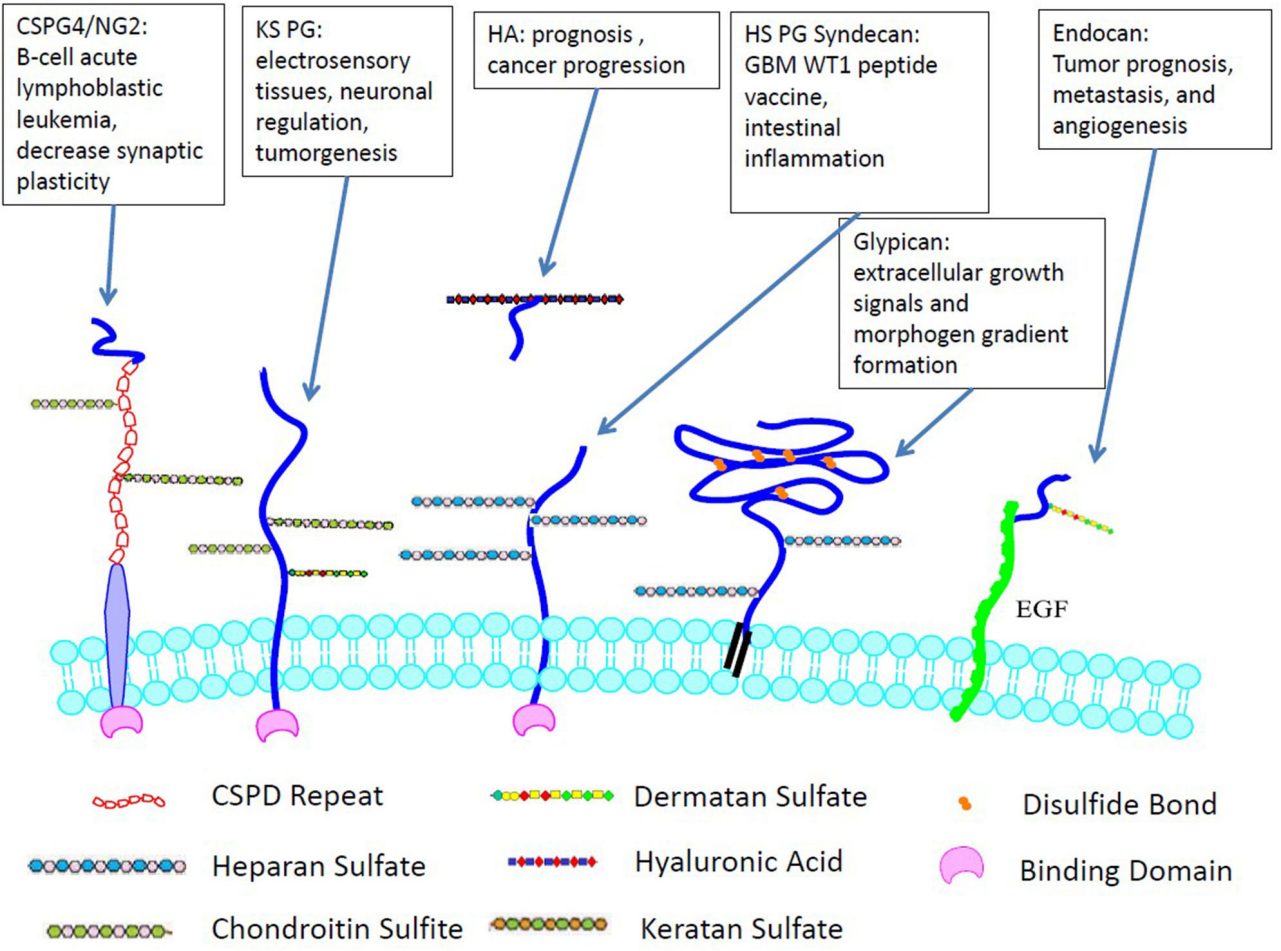
Cellular localization and significance for tumor development of GAGs. Unlike many other GAGs, HA is not covalently-linked to cell surface and only non-covalently interacts with PGs.

GAG to PG linkage is crucial to establishing and maintaining the fundamental functions of CNS, such as migration, cellular proliferation, specification, plasticity, synaptogenesis, and regeneration. Their mechanisms and functions have been summarized in many reviews (17–20). PG diversity depends on differential expression of protein sequences, variations in the length, and profile of GAG modifications. PGs regulate growth factors that affect cell adhesion, neurite outgrowth (21), ECM assembly, and tumor cell invasion (22, 23). Syndecans and glypicans are the two main transmembrane PGs containing HS chains in the CNS. Heparan sulfate PGs directly influence the aggregation and activity of AMPA receptors, which hinders cognitive functions by inducing or maintaining long-term potentiation (LTP) (24). Chondroitin sulfate PG, which is expressed abundantly in the cerebellum and hippocampus but decreases significantly postnatally, affects the stabilization of synapses and axonal sprouting (25). Figure 2 demonstrates the selected cellular localization and significance for tumor development of the PGs discussed.

### Chondroitin Sulfate Proteoglycan

Chondroitin sulfate PG 4, commonly referred to as neuron-glial antigen 2 (NG2), contributes to the stabilization of interaction between cell and substratum on endothelial basement membranes, especially at the early spreading stage of melanoma cells (26) In addition, CS PG 4 is a suggested biomarker in glioblastoma (GBM) (27). NG2-expressing oligodendrocyte precursor cells support neurons and synaptic signaling physiologically and carry out these functions in both brains that are healthy and those in the process of injury repair and regeneration. Moreover, NG2 protein facilitates tumorigenesis and tumor progression (28). In the previous study of human GBM cells, more cells survived under NG2-mediated activation (29) and chemoresistance through integrin-dependent PI3K/Akt signaling (30).

Gliomagenesis is induced by unusual expression of neuron-glial antigen 2 endocytosis *in vivo* murine oligodendrocyte precursor cells, which provides another mechanism through which benign precursor cells can be converted into cancer stem cells (31). Additionally, NG2-expressing precursor cells demonstrate significant developmental plasticity. For instance, activating Notch signaling induced pericyte-like differentiation in NG2-positive GBM cancer stem cells, which during tumor angiogenesis contributed to vessel stabilization (32). Although these results suggest that NG2 plays a role in cancer stem cells, it is still uncertain whether the GAG moieties or the PG's other functional domains are responsible for the stemness-related functions.

Lam et al. reported an efficient and effective “glial progenitor cell-based therapy” for congenital myelin CNS disorders (33). From bone marrow stromal cells, they produced glial progenitor cells in a 14-days CS PG 4-based induction protocol. The generated cells were highly enriched in oligodendrocyte precursor cell marker expression. After being transplanted into the myelin-deficient mice, the cells differentiated successfully into myelinogenic oligodendrocytes. Both lifespan and motor function were improved significantly by remyelination of the shiverer mouse. Their study demonstrated the feasibility of human bone marrow stromal cells as a source of glial progenitor cells for attaining such myelinogenic oligodendrocytes (33). The novel induction protocol overcame existing hurdles of cell source restriction and timeframe requirements, providing a method for efficient myelin disorder glial therapy.

In addition, lecticans were also investigated as a group of chondroitin sulfate PGs due to their role in linking ECM molecules (34). The unique composition of brain ECM causes brain tissue to resist invasion by non-neuronal tumors (35). Due to its moderate plasticity, CNS has a considerable capacity for regeneration, although changes in ECM have been observed after trauma and throughout the development of CNS disease. The modification of PGs in ECM is shown as one of the factors leading to change in CNS plasticity. Through control of neurotransmission and synaptic connections, the scaffold of proteins and sugars in the ECM changes the functionality of surrounding tissue (36).

To activate immune cells, CS PGs generally collect the microenvironment's signals and bind immunological receptors, thus boosting inflammatory responses. CS PGs also stimulate matrix-degrading enzymes and bind signaling molecules in immune cells such as chemokines and cytokines (37).

### Heparan Sulfate Proteoglycans

Heparin has anticoagulant activity and can only be produced by mast cells. Heparan sulfate also functions as an anticoagulant, though on a lower level than heparin, and is generated by nearly all cell types. Present both in the ECM and on the cell surface, heparan sulfate PGs (HSPG) facilitate cell-microenvironment interactions and cell signaling pathways. In GBM, HS glycosaminoglycans expression and their regulating enzymes are changed, but the structure and content of the HS itself remain unknown. For example, glypicans of the HS PG families [See review: Wang et al. (38)] are proteins that are membrane-bound and that modulate morphogen gradient formation and extracellular growth signals to engage in organ development (39). Crucially, some studies reported an increased level of glypicans in the peripheral blood of patients, holding glypicans as a promising new biomarker in the cancer field (40). Tran et al. used LC-MS analysis to portray the differences in both HS disaccharide content and structure. As a result, they suggested inter-tumoral differences in PG expression and function have potential implications for therapeutic stratification (41).

Spyrou studied the inhibition of heparanase in brain tumor cells of children and subsequently reduced their invasive capacity, proliferation, and tumor growth *in vivo*. The results suggest that heparanase affects both tumor cells and their ECM in cases of malignant brain tumors. However, the inhibitor (PG545) failed to pass the BBB due to its size, and thus direct injection or a new drug delivery system is required (42).

### Hyaluronic Acid

Hyaluronan (or hyaluronic acid) is a “multifunctional GAG synthesized as a large negatively charged linear polymer by distinct hyaluronan synthases” (43). While aggrecan-related components generally result in clear regional distribution patterns, hyaluronan is widely distributed in the white and gray matter (44). HA interacts with several cell membrane receptors, including CD44 and Lymphatic vessel endothelial hyaluronan receptor 1, the former being the more thoroughly studied receptor for HA-mediated motility in cancer progression. In addition, certain PGs use link modules to form supramolecular complexes with HA. Generally, high levels of HA and HA receptors are correlated with poor prognosis in cancer patients.

Using pluripotent stem cells-derived and primary brain microvascular endothelial cells, Al-Ahmad et al. tested the effect of HA on BBB properties. The impact of HA signaling on developmental and mature brain microvascular endothelial cells was assessed by measuring changes in transendothelial electrical resistance, permeability, brain microvascular endothelial cells markers localization, and expression, CD44 expression, and hyaluronan levels. HA treatment generally reduced barrier function and P-glycoprotein activity. The effects were more evident with treatment using oligomeric forms of HA and exacerbated when the treatment was applied during the brain microvascular endothelial cells differentiation phase (considered developmental BBB). The hyaluronidase activity, as well as an increase in CD44 expression during prolonged oxygen-glucose deprivation stress, were also observed. Inhibiting HA signaling by the antibody blockade of CD44 reversed the treatment's adverse effects, thus conveying the significance of HA signaling through CD44 on BBB properties (45). Moreover, Hartheimer et al. determined how hyaluronidases can sensitize GBM stem cells to chemotherapy drugs by disrupting the HA-CD44 signaling, with which they further developed a combined treatment of hyaluronidases and chemotherapy drugs by disrupting the stemness-promoting HA to target GBM stem cells. This combination therapy shows promise even when temozolomide treatment alone causes resistance (46).

### Dermatan Sulfate PG—Endocan: A New Biomarker and Therapeutic Target

Endocan is a novel endothelial cell-specific molecule with 50 kDa molecular weight and high solubility in water. As a proteoglycan, endocan is secreted into the blood and formed in the presence of CS. In normal tissues, CS and DS PGs are expressed in endothelial cells but are overexpressed in certain tumor endothelial cells. Unsurprisingly, abnormal expression levels of endocan were observed in tumor prognosis, angiogenesis, and metastasis. Researchers believe that the role of endocan is to regulate the tumor by tumor-related angiogenesis, cell inflammation, lymphangiogenesis, and other aspects (47). Accordingly, Kijima et al. studied surface marks from patient derived xenografts and cell lines based on array comparative genomic hybridization to investigate the early stages of GBM tumorigenesis (48). In additional to research that found raised levels of systemic inflammatory markers to be correlated with cardiovascular disease (49), a recent study revealed that the specific sulfation level of DS is crucial in synaptic plasticity and is related to changes in the expression of glutamate receptors and other associated synaptic proteins (50). As such, endocan became a valuable target for GBM diagnosis and therapy.

As another interesting target, dermatan sulfate epimerase 1 is overexpressed in many types of cancer as a tumor-rejection antigen. The CS/DS chains mediate several growth factor signals. However, investigating their roles in gliomas involves less work. Liao et al. examined the expression of Dermatan sulfate epimerase 1 in gliomas by utilizing a public database and conducting immunohistochemistry on a tissue array. Their investigation revealed that Dermatan sulfate epimerase 1 regulates the HB-EGF/ErbB pathway, which participates in GBM cells' malignant behavior. Treating epidermal growth factor receptor and ErbB2 with selected inhibitors thus suppressed malignant phenotypes, demonstrating that the upregulation of Dermatan sulfate epimerase in gliomas contributes to controlling malignant behavior in cancer cells (51).

### Keratan Sulfate (KS)

Keratan sulfate (KS) is a sulfated GAG, which contains structurally unique characteristics of diversity in the linker oligosaccharides connecting to the core protein. The repeating disaccharide unit in KS contains one galactose and one N-acetylglucosamine and is linked to core proteins via either N-linked or O-linked glycosylation of the PGs. KS is most abundant in the cornea, and second abundant in the brain (52). Negatively charged KS modifications of synaptic vesicle protein 2 interacted with both Ca^2+^ ions and other neurotransmitters such as dopamine, establishing the PG delivery complex (53). Furthermore, high sulfation level KS PGs are commonly found in the brain. For example, synaptic vesicle proteins 2 played significant neuronal and synaptic regulatory roles (54).

An earlier study reported that highly sulfated KS was overexpressed in malignant astrocytic tumors (55). It has also been found that the interruption of Synaptic vesicle proteins 2 functionality is associated with epilepsy (56). These results were confirmed by the subsequent research of the interactivity of KS with nerve growth factor and receptor proteins, neuroregulatory proteins, synaptic proteins and neurotransmitters (57). In addition, abnormal sulfation degrees of KS are observed in the brains of Alzheimer's patients (58). Tsidulko's work demonstrated that PG composition and ECM structure in normal brain tissue were affected during temozolomide induced chemotherapy. These changes were believed to participate in the development of the tumorigenic niche for the expansion of the residual glioma cells and the disease progression (59). Recently, researchers have reviewed the influences of KS sulfation on electrosensory tissues and neuronal regulation. KS with overexpressed sulfation level interacts with neuroregulatory proteins. Hence, actin and tubulin cytoskeletal development was stabilized by KS PG microtubule-associated proteins during neuritogenesis (60).

## The Role of PGs in Brain Inflammation And Plasticity

In general, PGs have influenced several aspects of tumor biology such as tumor cell adhesion and migration, cell proliferation, angiogenesis, and inflammation. Up- and down-regulated expression in PG core proteins is observed in many cancers and usually related to changes in cell signaling and invasion (19, 61). Jang et al. found that the interaction between the intracellular domain of some transmembrane PGs with the cytoplasmic domain of proteins promoted the signaling (62). In an earlier review, the cytoplasmic domain of syndecan-1 was found to have interaction with talin to modulate integrin signaling via a syndecan-1-integrin-insulin-like complex (63). Likewise, the cytoplasmic tail of transmembrane heparan sulfate PG syndecan-4 interacts with α-actinin regulating cytoskeletal organization. Fröhling et al.' recent research suggested that the loss of syndecan-4 expression is correlated with the increase if intestinal inflammation. While primarily expressed in the colonic epithelium, syndecan-4 accumulated the deficiency during the growth of susceptibility regarding the intestinal inflammation. Mechanisms were proposed that syndecan-4 played a role in protecting against inflammation, keeping the epithelial gut barrier's unity and regeneration (64). When using anti-syndecan-4 antibodies as a therapeutic approach to treat patients with inflammatory disorders, researchers must carefully evaluate patients who have inflammatory diseases associated with an epithelial barrier function.

Many PGs were proposed as markers for therapy evaluation. For example, syndecan-4 mRNA expression was specified as the unique marker to predict the GBM multiforme patient's response during the WT1 peptide vaccine treatment (65). Letoha et al. reported that syndecan-4 bound and mediated the transfer of a cell-penetrating short peptide with 17 amino acids into the cells (66). Roy et al. then demonstrated a positive correlation between glioma grade and serglycin expression level in GBM progression (67).

There is significant evidence showing that the sulfate composition of CS GAG chains changes with age. As a result of aging and aggregation of proteins, the deposition of HS PGs and CS PGs results in the injury of protective perineuronal nets with increased cell death (60). Dying neurons then induce inflammation, ECM degrades through the proteolytic activity of enzymes, inducing responses that amplify neuronal death and neuroinflammation (68). Simultaneously, overexpression of chondroitin 6-*O*-sulfotransferase 1 may decrease the ratio of 4–6S in perineuronal nets and increase seizure susceptibility (69). This is supported by Foscarin's work that the age-associated rise in the ratio of 4–6S GAG in perineuronal nets may decrease synaptic plasticity (70). Their studies highlighted the necessity for genetic manipulation of other enzymes such as chondroitin sulfotransferases to discover their biological functions and generate the profile of sulfation's role in development and aging.

Recent preclinical research demonstrated the antitumoral effects of chondroitin sulfate PG 4. The NG2-directed chimeric antigen receptor T-cells were proved to efficiently target GBM cancer stem cells (71). The combination of anti-NG2 antibodies was induced in chemotherapy in B-cell acute lymphoblastic leukemia (72).

## Conclusion

In summary, adjustments in PG core proteins, biosynthetic enzymes, and extracellular regulating enzymes are correlated with many developmental anomalies and overgrowth or tumor predisposition syndromes. PGs facilitate the activity of various signaling pathways and stimulate cell-microenvironment interactions in tumors. Due to such a diverse range of functions, PGs and their modifying enzymes are an imperative area of study that may potentially uncover therapeutic targets and biomarkers of GBM. In the damaged CNS, PGs accumulate during traumatic brain injuries, multiple sclerosis, and spinal cord injuries, driving pathogenesis and neuroinflammation. It should be noted that compared to the *in vitro* examination, more complex factors may interfere with the regulated expression of PGs in ECM *in vivo*. When assessing experiment data for therapeutic targets and treatment strategies, researchers should carefully consider adverse side effects that can be avoided in advance.

## Author Contributions

All authors participated in writing and revising the manuscript.

## Conflict of Interest

The authors declare that the research was conducted in the absence of any commercial or financial relationships that could be construed as a potential conflict of interest.

## Footnotes

^1^Available online at: https://www.cancer.org/content/dam/cancer-org/research/cancer-facts-and-statistics/annual-cancer-facts-and-figures/2019/cancer-facts-and-figures-2019.pdf

## Abbreviations

BBB: blood-brain barrier
CS: chondroitin sulfate
CNS: central nervous system
DS: dermatan sulfate
ECM: extracellular matrix
GAGs: glycosaminoglycans
GBM: glioblastoma
HS: heparan sulfate
HP: heparin
HA: hyaluronic acid
KS: keratan sulfate
NG2: neuron-glial antigen 2
PG: proteoglycan.
